# The number of tumorspheres cultured from peripheral blood is a predictor for presence of metastasis in patients with breast cancer

**DOI:** 10.18632/oncotarget.10174

**Published:** 2016-06-20

**Authors:** Monika Pizon, Dorothea Schott, Ulrich Pachmann, Katharina Pachmann

**Affiliations:** ^1^ Transfusion Center Bayreuth, 95448, Bayreuth, Germany

**Keywords:** breast cancer, circulating epithelial tumor cells, circulating cancer stem cells, predictor for presence of metastasis

## Abstract

**Background:**

Tumor metastases are the major cause of cancer morbidity and mortality. A subpopulation of tumor cells with stem-like properties is assumed to be responsible for tumor invasion, metastasis, heterogeneity and therapeutic resistance. This population is termed cancer stem cells (CSCs). We have developed a simple method for identification and characterization of circulating cancer stem cells among circulating epithelial tumor cells (CETCs).

**Methods:**

CETCs were cultured under conditions favoring growth of tumorspheres from 72 patients with breast cancer, including a subpopulation of 23 patients with metastatic disease. CETCs were determined using the maintrac® method. Gene expression profiles of single CETCs and tumorspheres of the same patients were analyzed using qRT-PCR.

**Results:**

Sphere formation was observed in 79 % of patients. We found that the number of tumorspheres depended on stage of disease. Furthermore, the most important factor for growing of tumorspheres is obtaining chemotherapy. Patients with chemotherapy treatment had lower numbers of tumorspheres compared to patients without chemotherapy. Patients with HER2 positive primary tumor had higher number of tumorspheres. Analysis of surface marker expression profile of tumorspheres showed that cells in the spheres had typical phenotype of cancer stem cells. There was no sphere formation in a control group with 50 healthy donors.

**Conclusions:**

This study demonstrates that a small fraction of CETCs has proliferative activity. Identifying the CETC subset with cancer stem cell properties may provide more clinically useful prognostic information. Chemotherapy is the most important component in cancer therapy because it frequently reduces the number of tumorspheres.

## INTRODUCTION

Breast cancer is the most common cancer in women worldwide and development of distant metastases is a main reason for cancer mortality. Distant metastasis formation is due to spread of cancer cells from the primary location through circulation [1, 2]. Presence of circulating tumor cells was first demonstrated in 1869 [3] and their existence in the blood have been associated with metastasis formation. Enumeration, longitudinal monitoring and characterization of circulating tumor cells can contribute to individually tailor therapy, improve design of personalized therapies, monitor treatment efficiency, enhance prognostic accuracy and advance our understanding of the biology of metastatic disease [4, 5, 6]. Recently the hypothesis has been proposed that a more aggressive subpopulation of circulating tumor cells, circulating cancer stem cells (cCSCs), are the source of metastatic spread from the primary tumor [6, 7]. The presence of cancer stem cells (CSCs) in neoplastic tissue has been a long standing hypothesis, and recently, these cells have first been identified in leukemia and subsequently in different solid tumors [8, 9]. CSCs carry typical stem cells properties; they are capable of undergoing extensive proliferation and self-renewal through asymmetric division and differentiation into non-tumorigenic cancer cells. Furthermore CSCs have been found to have increased resistance to chemotherapeutic agents and radiation [10, 11]. CSCs have been identified and isolated from solid tumor tissue or cancer cell lines by different methods such as CSC-specific cell surface marker expression and aldehyde dehydrogenase (ALDH1) activity and their ability to grow as floating spheres (tumorspheres) [12, 13]. Multiple markers including CD44, CD24 and EpCAM are used to identify breast cancer stem cells. Up-regulation of the expression of CD44 a marker for stem cells of several types of cancer and the major hyaluronan receptor increases tumor growth and has an anti-apoptotic effect [13, 14]. CD24 has been investigated in combination with CD 44 or other surface antigens in various cancers [13, 15]. The overexpression of the Epithelial Cell Adhesion Molecule (EpCAM) enhances the proliferative and invasive capacities of tumors [16]. Aldehyde dehydrogenase 1 (ALDH1) is a detoxifying enzyme responsible for oxidation of retinol to retinoic acid and may have a role in early differentiation of stem cells [13, 17].

Transcription factors such as Oct4, Sox2 and Nanog are required to maintain pluripotency and self-renewal capacity of cancer stem cells and play an important role in the uncontrolled proliferation of cancer cells [18].

A functional approach, tumorsphere formation assay, based on the unique property of stem cells to survive and grow in suspension culture, is successfully applied to enrich stem cells from invasive tumor samples or cancer cell lines. Tumorspheres have been reported to display typical stem cell surface markers like CD44^+^/CD24^−^, overexpress neoangiogenic and cytoprotective factors and genes of pluripotency [19, 20].

The aim of the present study was to develop a new approach based on functional, pheno-and genotypic features of CSCs for detection and characterization of cells with proliferative activity and cancer stem cell properties among circulating epithelial tumor cells.

## RESULTS

Patients characteristics are given in Table 1. Among the 72 patients there were 49(68%) patients with non-metastatic and 23 (32%) with metastatic disease. The primary tumors were histologically positive for ER and PR in 44 (71%) patients, and positive for HER-2/neu in 6 (11%) patients.

**Table 1:** Clinico-pathological characterisitcs in breast cancer patients

Number of patients (frequency)		
**Age (years)**		
	<40	2 (2.7%)
	40-49	18 (25%)
	50-59	27 (37.5%)
	60-69	17 (23.6%)
	70+	8 (11.2%)
**Stage of disease**		
	I	18 (25%)
	II	17 (24%)
	III	14 (19%)
	IV	23 (32%)
**Axillary lymph node**		
	Positive	31 (43.1%)
	Negative	37 (51.4%)
	unknown	4 (5.5%)
**Metastasis**		
	Present	23 (31.9%)
	Absent	49 (68.1%)
**HER2 status**		
	Positive	6 (8%)
	Negative	54 (75%)
	unknown	12 (17%)
**ER/PR status**		
	Positive	44 (61%)
	Negative	18 (25%)
	unknown	10 (14%)
**Chemotherapy**		
	Neoadjuvant	3 (4.2%)
	Adjuvant	22 (30.5%)
	without chemotherapy	41 (56.9%)
	unknown	6 (8.4%)

**Figure 1:**
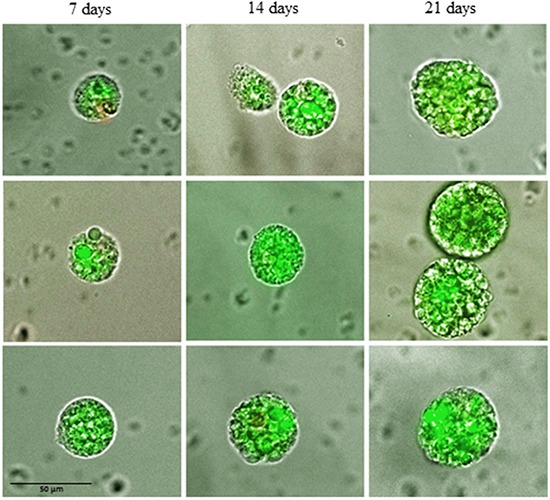
Changes in size of typical tumorspheres after 7, 14 and 21 days of culture

During culture in non-adhesive, suspension culture the CETCs with proliferative activity grew as spherical tumorspheres. After 3-5 days of culture first signs of sphere formation were observed. Spheres grew gradually over time and after 21 days of culture all spheroids had a diameter between 30 and 60 μm (median 40) (Figure 1). For cultivation of tumorspheres we included patients with cell numbers ranging from 0 to 2860 CETCs/100μl blood. The fraction of tumorspheres varied between 0-10% of the CETCs. We found no statistically significant correlation between the number of CETCs and tumorspheres (p=0.7) indicating that the number of spheres is independent from the number of single circulating tumor cells. Interestingly, in two cases of patients with metastatic breast cancer without EpCAM positive CETCs we observed more than 30 tumorspheres/100μl blood. In contrast, no spheres could be grown from blood of 50 normal subjects. To test for self-renewal capacity established tumorspheres were dissociated into single cells, plated and the number of secondary spheres that formed after 14 days counted. Approximately, 15% to 20% of the tumorsphere cells gave rise to secondary spheres indicating that the tumorspheres contain self-renewing stem cells.

**Figure 2:**
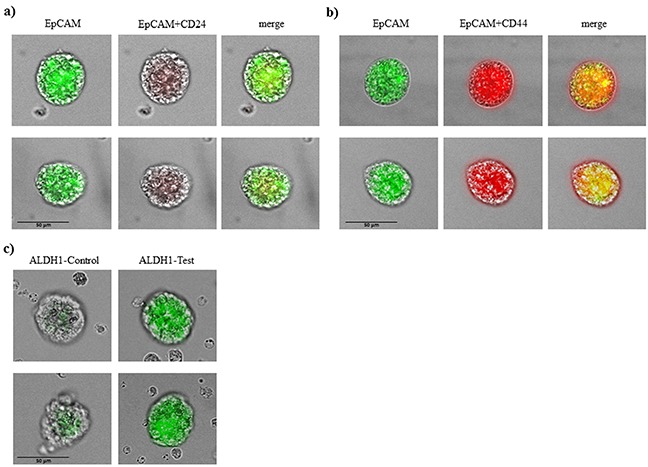
Immunophenotypic analysis of tumorspheres in breast cancer patients **A.** tumorspheres are low positive or negative for CD 24 and **B.** positive for CD44 and EpCAM. **C.** tumorspheres had also elevated level of ALDH1 activity.

Counterstaining for specific stem cell markers is shown in Figure 2a, 2b. Tumorspheres in patients with breast cancer were positive for EpCAM and also for CD 44 and negative or low positive for CD 24. All tumorspheres showed distinct fluorescence for ALDH1 (Figure 2c) typical for breast cancer stem cells.

**Figure 3:**
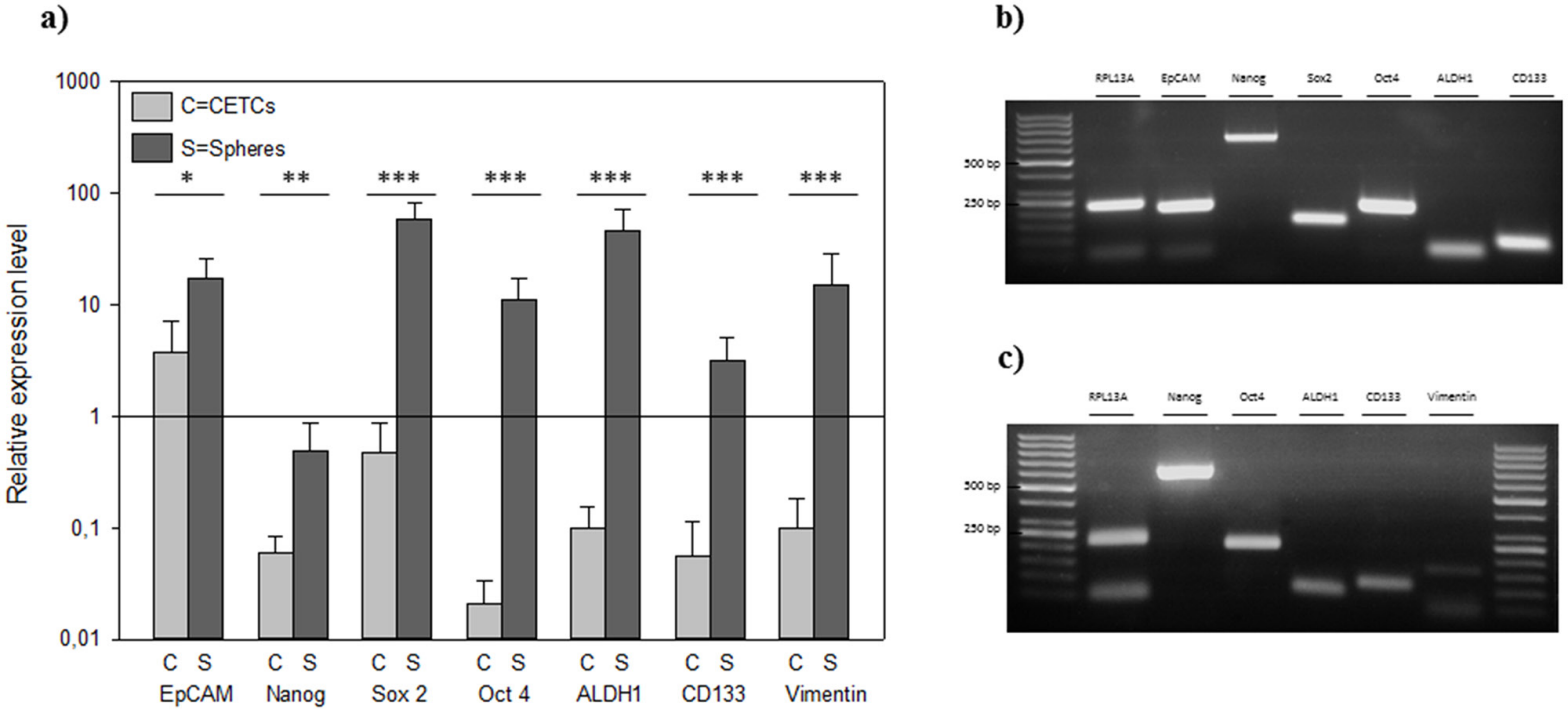
**A.** Relative expression levels of typical cancer stem cell genes in individual CETCs and tumorspheres from the same patient. Gel picture of the qRT-PCR assay of **B.** single tumorsphere and **C.** single CETC.

The expression levels of the putative stem cell markers Oct4, Sox2, Nanog, EpCAM, ALDH1 and CD133 showed overexpression in relation to house-keeping gene RPL13a in tumorspheres whereas the expression level of these stem cell markers was significantly lower in CETCs (p<0.05) (Figure 3).

**Figure 4:**
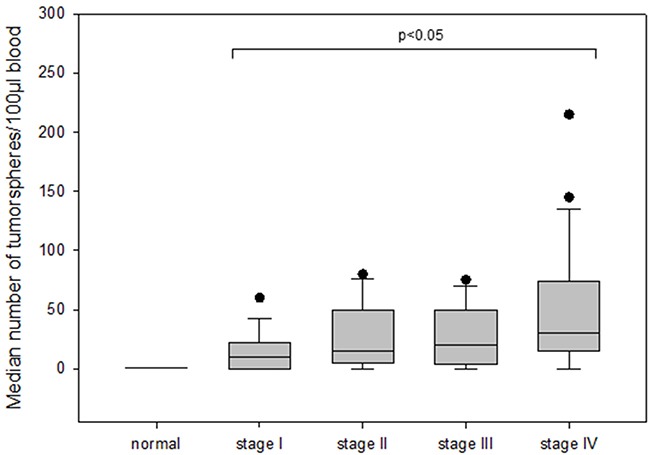
The number of tumorspheres in different stages of disease (I-IV) in breast cancer patients

**Figure 5:**
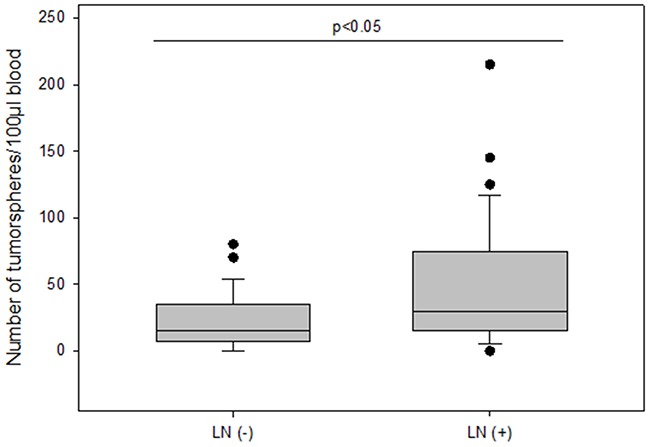
The number of tumorspheres without and with axillary lymph node metastasis

The number of tumorspheres increased with stage of disease and patients in stage IV had the highest number of tumorspheres differing significantly from patients in stage I (median 30 vs 10; p=0.002) (Figure 4). Furthermore, we found that patients with positive axillary lymph node status possessed significantly more tumorspheres as compared to patients with negative lymph nodes (median 30 vs 15; p<0.05) (Figure 5).

**Figure 6:**
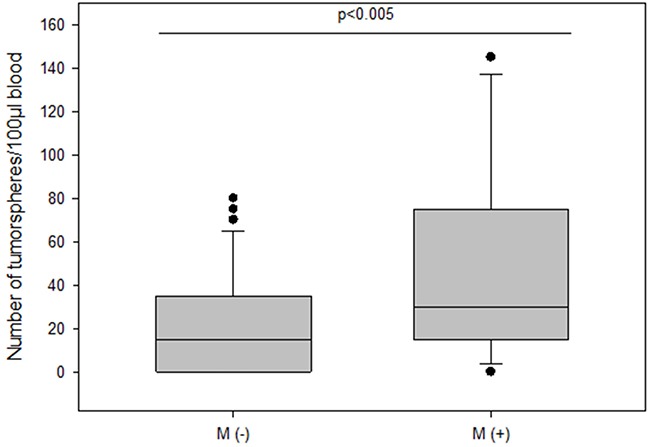
The number of tumorspheres in non-metastatic and metastatic patients

**Figure 7:**
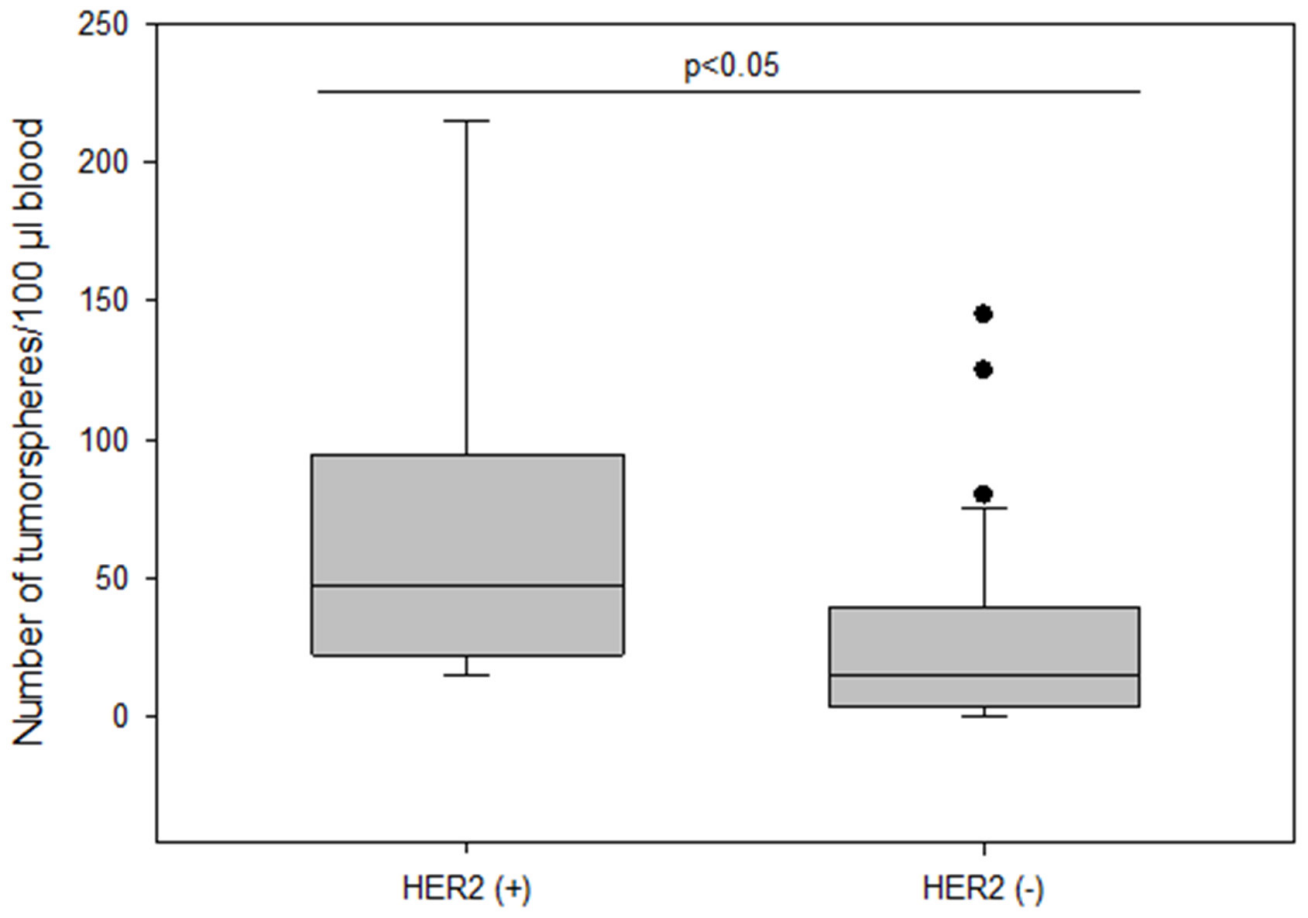
The number of tumorspheres and HER2 status in primary tumor

The frequency of spheroids cultured from CETCs was monitored in patients without and with distant metastases in order to test whether the number of tumorspheres correlated with disease progression. The total amount of tumorspheres was higher in patients with metastatic disease as compared to patients without metastases (median 30 vs 15; p<0.005) (Figure 6). Patients with HER2 positive primary tumors had the highest numbers of tumorspheres with a median of 50 (Figure 7). Thus, our results indicate that the frequency of cells from the subpopulation of CETCs that can grow into tumorspheres correlates with the aggressiveness of the tumor.

**Figure 8:**
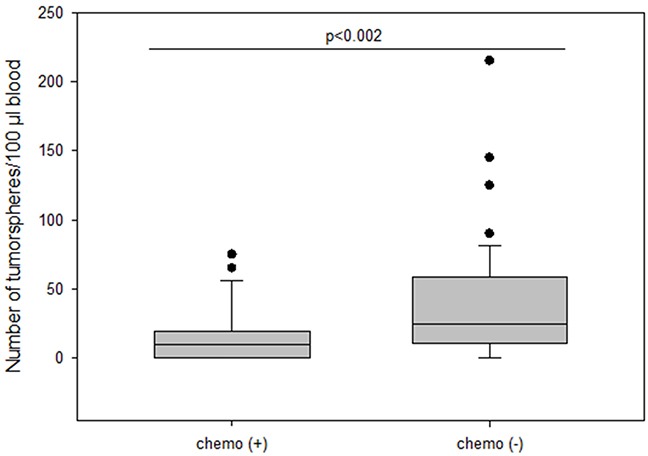
The number of tumorspheres in patients with and without chemotherapy

**Figure 9:**
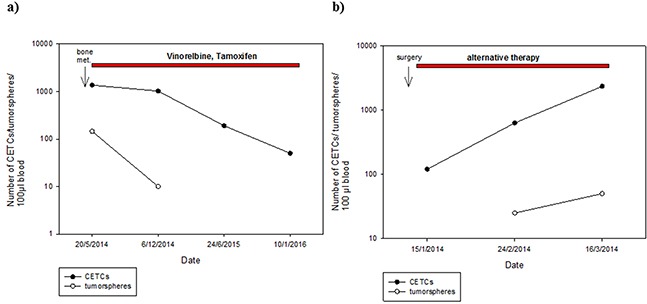
Exemplary courses of CETC and tumorsphere numbers from two individual patients with breast cancer **A.** metastatic breast cancer patient (bone metastases) with decreasing CETC and tumorsphere numbers during therapy with Vinorelbine and Tamoxifen **B.** increasing CETC and tumorsphere numbers in breast cancer patient (stage III) without cytotoxic treatment.

Surprisingly, patients who received no chemotherapy (56.9%) had higher numbers of tumorspheres compared to patients who had received chemotherapy (34.7%) (median 25 vs 10; p=0.002) (Figure 8). Moreover, numbers of tumorspheres were shown to vary depending on chemotherapy. Figure 9a, 9b shows two representative serial analyses of CETCs and tumorspheres in two exemplary breast cancer patients. In Figure 9a) the course of decreasing CETCs and tumorspheres during chemotherapy is shown. Figure 9b) shows increasing CETCs and tumorspheres in a patient without cytotoxic treatment.

**Figure 10:**
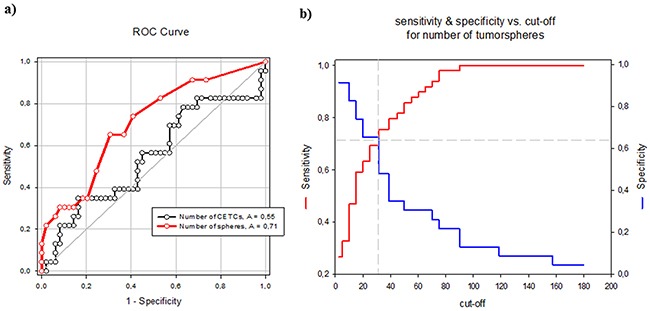
**A.** ROC curves for tumorsphere and CETC count to discriminate metastatic and non-metastatic breast cancer patients. **B.** Sensitivity and specificity values at selected cut-off points for number of tumorspheres.

In contrast to the number of CETCs the number of tumorspheres was an independent factor of the presence of metastases (Odds Ratio 1.1; Coefficient 0.119; p=0.008) in patients with breast cancer. The receiver operating characteristic (ROC) curve shows that the number of spheres that can be grown from a patient's blood is a predictor of the presence of distant metastases. The AUC for number of spheres was higher than for the number of CETCs and it is 0.71 (p=0.005) (Figure 10). The cut-off number of spheres predictive of metastases was 30/100μl blood with a sensitivity of 70% and a specificity of 65%.

## DISCUSSION

**Figure 11:**
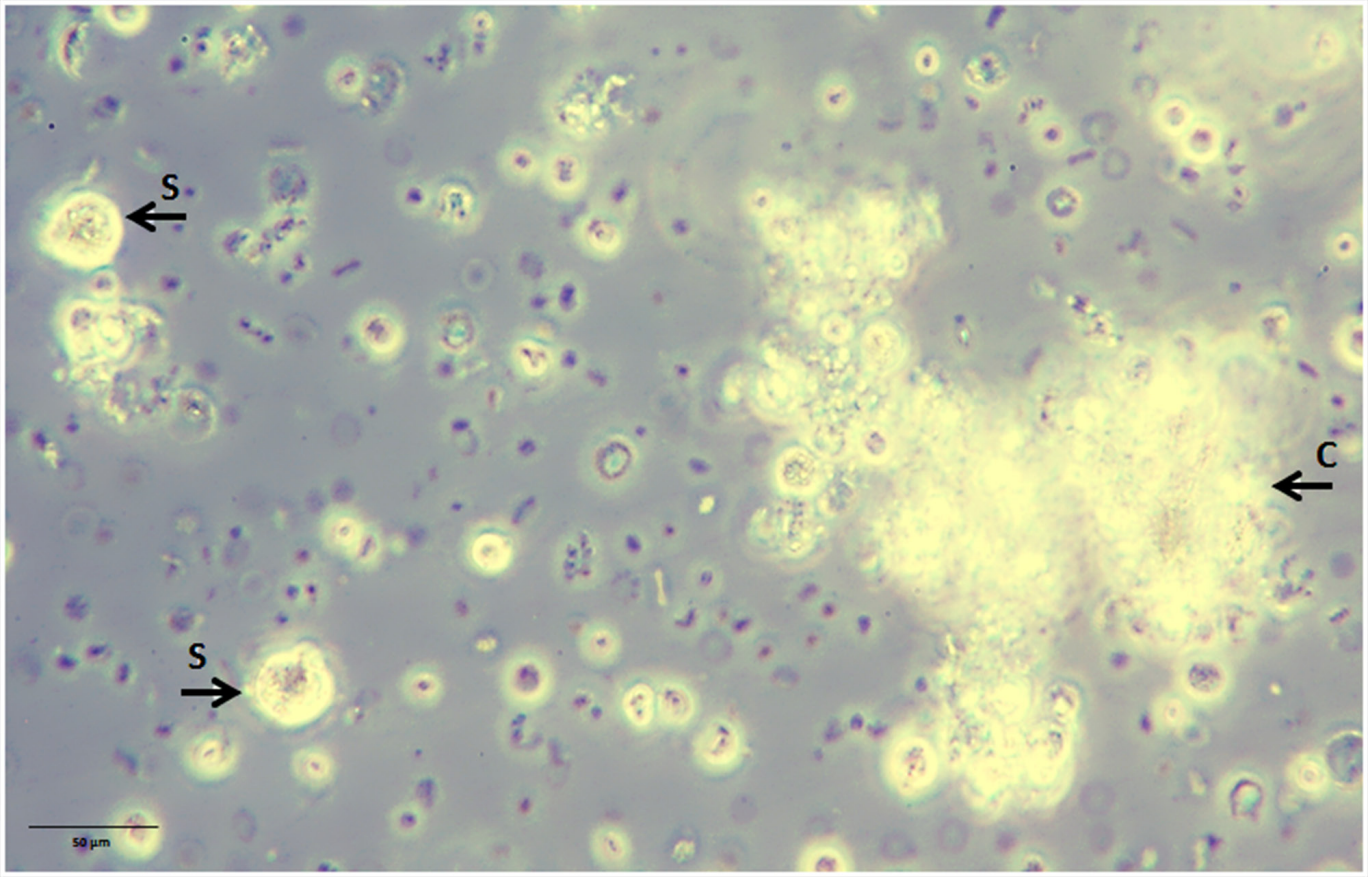
Non-adherent tumorspheres developed in suspension culture under stem cell-like growth conditions Bright smooth-edged and compact spheres (S) reached 20 μm after 3 days of culture. Leucocytes aggregate to each other and form clumps (C) with an irregular outline.

Culturing blood white cells comprising CETCs from patients with breast cancer we were able to identify a subpopulation of CETCs with clonal proliferative activity growing into tumorspheres. These tumorspheres can be cultured in special medium supplemented with growth factors during 21 days at 37°C and 5% CO_2_ in conventional cell culture flasks. Only a small subpopulation of epithelial tumor cells circulating in patients' blood had the capacity to form spheres under these conditions and no spheres could be grown from normal subjects. In contrast to Yu et al we were able to successfully culture CETCs from breast cancer patients in early stage as well as in advanced stage of disease [22]. Sphere-forming efficiency may be due to autocrine/paracrine signals released by accompanying cells into the medium [23]. In our experiment morphologic and immunohistochemical analysis show that spheroids cultured from circulating tumor cells are bright, smooth edged and compact and are clearly different from irregular clumps of cells that also appeared at times in the culture (Figure 11). Furthermore, cultivation is performed without movement of culture flasks and leukocytes are dying during cell culture. Finally, we observed no sphere formation in healthy objects (negative control) which excludes the formation of tumorspheres as a result of aggregation of white blood cells.

Based on the type of growth, staining properties and genotypic characterization of the spheres this subpopulation had cancer stem cell (CSC) properties and these cells may be progenitors of metastases leading to relapse and treatment failure in patients with breast cancer.

Immunostaining showed both CD44 high/CD24 low expression and ALDH1 activity which are CSC markers. CD44 normally takes part in cell-cell and cell-matrix adhesion interactions, which is involved in cancer cell migration, proliferation and metastasis. Current experimental evidence indicates that the CD24 negative subpopulation might represent a more drug resistant phenotype [24]. It has been shown that breast cancer stem cells present high levels of ALDH1 [16]. Our findings are consistent with these results.

The expression of pluripotency- associated factors playing a crucial role in the development and malignant progression of various types of cancer including breast cancer [25], Aktas et al. could show that cells enriched for tumor suspected cells from metastatic breast cancer patients exhibited mRNA expression of ALDH1. These indirect indications from expression profiles led them to assume that a subpopulation among the very heterogenic circulating tumor cells display cancer stem cell characteristics [26]. We, here, demonstrate that CSC can be directly visualized by forming tumorspheres and Nanog, SOX2, OCT4, ALDH1, CD 133 and EpCAM was elevated in these tumorspheres relative to CETCs. Enumeration of tumorspheres strongly indicated that the number of spheres correlates with the aggressiveness of the tumor, e.g. lymph node involvement and metastasis. Patients with stage I had significantly less tumorspheres as compared to patients with distant metastasis. Comparing patients with and without metastatic disease revealed a tumorsphere count ≥ 30 spheres/100 μl blood as an independent predictor of the presence of metastatic disease (AUC-ROC, 0.72; p<0.05), growth of more than 30 spheres/100μl blood in patients with diagnosis of breast cancer warranting additional diagnostic imaging for possible distant metastases. As already evidenced in some studies dynamics of the number of CETCs is more important and prognostically relevant than single cell count analysis [4, 27, 28]. Single cell counting per se does not reflect the aggressiveness of tumor burden, especially in cases after surgery which leads to a temporary increase in cell numbers [29].

The number of tumorspheres was significantly higher in patients with HER2 positive tumors compared to HER2 negative tumors consistent with the finding of Korkaya et al. that HER2 overexpression increases the mammary stem cell population and also increases the capacity for sphere formation from tissue cultures. Furthermore, overexpression of HER2 in a series of human cancer cell lines increases the percentage of cells with ALDH activity, leading to increased amounts of spheres [30] suggesting that HER2 overexpression plays an important role in tumor growth, invasion and metastasis.

In the adjuvant situation where there is no residual disease present monitoring of CETCs has been shown to provide a valuable tool to routinely monitor response to therapy [4] and is currently the only available approach. We, here, show that as a sign of successful therapy also number of tumorspheres can decrease concurrently with CETCs during chemotherapy. In contrast, increasing numbers of CETCs and tumorspheres might warrant further diagnostic steps in order to timely change or initiate therapy. Knowing that not all patients benefit from chemotherapy, some patients might want to avoid chemotherapy. Our data, however, show that patients receiving no chemotherapy after surgery have increased levels of tumorspheres. Monitoring therapy using CETCs and spheroids might lead to new treatment considerations in personal tailoring of therapy. Finally, we found no relationship between the number of CETCs and presence of metastasis. The growth of tumorspheres was independent of the number of EpCAM positive circulating cells. So far, we do not know from which progenitor cells the tumorspheres arise because in two patients without EpCAM positive circulating cells we still observed EpCAM positive tumorsphere formation. It is extensively described that the phenotypical change of cancer cells known as epithelial-mesenchymal-transition (EMT) is a critical step in tumor invasion and metastasis. During this transition the expressions of epithelial markers such as EpCAM and cytokeratin is assumed to be downregulated and circulating tumor cells may become undetectable [31]. For this reason our new approach enables, without the necessity to rely on any markers and thus without selection and loss of cell populations, the identification of the most important subpopulation of CETCs with proliferative capacity and clonal expansion.

In conclusion, we are able to culture a subpopulation of circulating tumor cells and pheno- and genotypically characterize them for putative stem cell markers. We postulate that tumorspheres are a surrogate of distant metastasis in breast cancer patients. Further studies could provide new insights into the tumorigenic process and assess the potential of these spheroids in breast carcinomas as therapeutic target.

## MATERIALS AND METHODS

**Figure 12:**
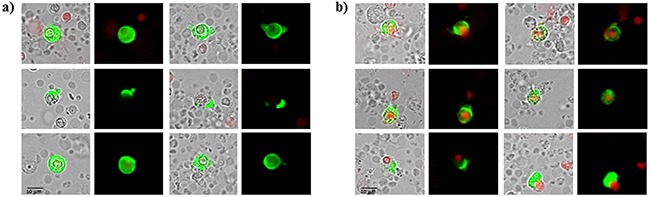
**A, B.** Examples of live and dead CETCs. A) Live CETCs have intact morphology with a well preserved membrane without PI staining in the nucleus. B) Dead CETCs show a positive PI staining because of loss of membrane integrity.

72 patients with histologically confirmed breast cancer, 49 with primary breast cancer and 23 patients with metastatic disease were included in the study. Blood samples were drawn into normal blood count tubes with ethylene diaminetetraacetic acid (EDTA) for enumeration and cultivation of CETCs. The maintrac® approach was used for detection and quantification of CETCs, as reported previously [21]. In brief, 1 ml blood was subjected to red blood cell lysis using 15 ml of erythrocyte lysis solution (Qiagen, Hilden, Germany) for 15 min in the cold, spun down at 700 g and re-diluted in 500 ml of PBS-EDTA. 5μl of fluorescein-isothiocyanate (FITC)-conjugated mouse anti-human epithelial antibody (EpCAM) (Miltenyi Biotec GmbH, Germany) was added and incubated for 15 min in cold. Subsequently, the samples were diluted with 430 μl PBS-EDTA and stored over night at 4°C. A defined volume of the cell suspension and propidium iodide (Sigma-Aldrich, USA) was transferred to 96-wells plate (Greiner Bio-one, USA), evaluated with a fluorescence scanning microscope (ScanR, Olympus, USA) and visually examined for presence of fluorescence and cell morphology. Only vital CETCs were counted with positive EpCAM fluorescence, lacking of nuclear PI staining and with intact morphology (Figure 12). We used two types of quality controls for ensuring the consistent analysis of samples. Isotype control, as negative control to measure the level of non-specific background signal and fluorospheres (Flow-Check 770, Beckman Coulter, USA) for daily verification of optical components and detectors. The percentage of circulating cancer stem cells present in the population of circulating epithelial tumor cells was determined by observing the number of cells capable to clonally grow into tumorspheres: after erythrocyte lysis and one centrifugation step CETCs together with leukocytes were plated at a density of 2×10^5^ cells/ml in RPMI-1640 medium supplemented with L-glutamine, HEPES, penicillin/streptomycin and growth factors such as EGF, insulin and hydrocortisone. Cells were maintained at 37°C and 5% CO_2_ without movement of the culture flasks. Fresh medium was added every five days and formation of tumorspheres was observed under an inverted light microscope (Primo Vert, Zeiss, Germany) at 40x magnification every 7 days. After 21 days tumorspheres were collected and prepared for immunostaining. Samples were centrifuged at 250 g for 7 min. The supernatant was discarded and the pellet resuspended with 500 μl PBS. 50 μl of cell suspension was transferred into 1.5 ml reaction tubes and prepared for the phenotypic characterization. Tumorspheres were incubated with 5 μl of fluorescein-isothiocyanate (FITC)-conjugated mouse anti-human EpCAM antibody (Miltenyi Biotec GmbH, Germany) and 5 μl of phycoerythrin (PE)-conjugated mouse anti-human CD-44 antibody (BD Bioscience, USA) or with 5 μl of phycoerythrin (PE)-conjugated mouse anti-human CD-24 antibody (BD Bioscience, USA) for 15 min in cold. The samples were subsequently diluted with 430 μl PBS-EDTA and then stored overnight at 4°C. 100 μl of cell suspension was transferred to 96-wells plates (Greiner Bio-one, USA). Analysis of red and green fluorescence of the tumorspheres was performed using a fluorescence scanning microscope (ScanR Olympus, USA). Tumorspheres were stained with propidium iodide to evaluate their viability prior to analysis. Finally, only vital tumorspheres with intact morphology were counted. ALDH1 activity of spheroids was determined using an ALDEFLUOR assay kit (Stem Cell Technologies^TM^, Canada) according to the manufacturer's protocol. In short, tumorspheres were suspended in ALDEFLUOR assay buffer containing ALDH1 substrate and incubated 45 min at 37°C. In parallel, a sample was treated with an ALDH1-specific inhibitor, as negative control for background fluorescence. Stained spheroids were analyzed with fluorescence scanning microscope (ScanR Olympus, USA). Moreover we examined 50 healthy donors aged from 20 to 53 years for determination of specificity and sensitivity and we observed no sphere formation in all cases.

In some cases we performed dissociation of spheroids with StemPro®Accutase® (Gibco, USA) for 3 min at room temperature to obtain a second generation of tumorspheres.

For quantitative RT-PCR CETCs and tumorspheres were isolated individually using a semi-automated capillary approach and deposited one by one into micro cups. cDNA was prepared with CellAmp^TM^ Whole Transcriptome Amplification kit (Takara Bio Inc./Mobitec). Subsequently, PCR was carried out with Light Cycler® 480 SYBR Green I Master (Roche). The following primers were used in this study: *rpl13a*: AGC TCA TGA GGC TAC GGA AA (forward) and CTT GCT CCC AGC TTC CTA TG (reverse); *epcam*: GGG AAA TAG CAA ATG GAC ACA (forward) and CGA TGG AGT CCA AGT TCT GG-5 (reverse); *vimentin:* TCC GCA CAT TCG AGC AAA GA (forward) and ATT CAA GTC TCA GCG GGC TC (reverse); *nanog:* GGA TCC AGC TTG TCC CCA AA (forward) and TGC ACC AGG TCT GAG TGT TC (reverse); *oct4:* GGC CAC ACG TAG GTT CTT GA (forward) and ATA CCT TCC CAA ATA GAA CCC C (reverse); *sox2:* GCG GAA AAC CAA GAC GCT C (forward) and TCA TGT GCG CGT AAC TGT CC (reverse); *cd133:* GTC CTG GGG CTG CTG TTT AT (forward) and TCT GTC GCT GGT GCA TTT CT (reverse); *aldh1* CTG AGC CAG TCA CCT GTG TT (forward) and GGA CAG GTA AGT CTG GCG TG (reverse). Relative expression levels were calculated after normalization to the reference gene ribosomal protein L13a (*RPL13a)* by using the ΔΔ*C*T method.

Data were analyzed using Student's t test for comparison of two variables or ANOVA for three and more variables. If the sample distribution was asymmetrical nonparametric tests (Mann Whitney U test for comparison two groups and Kruskal-Wallis for more groups) were performed. Relationships between parameters were evaluated using Pearson or Spearman's rank correlation.

Diagnostic performance of tumorsphere count was assessed by constructing a ROC curve, and was evaluated by calculating the area under the ROC curve.
